# Total Posterior Spinal Arthroplasty Systems for Dynamic Stability

**DOI:** 10.7759/cureus.12361

**Published:** 2020-12-29

**Authors:** Brian Fiani, Christian Noblett, Daniel Chacon, Imran Siddiqi, Elisabeth Pennington, Michael Kortz

**Affiliations:** 1 Neurosurgery, Desert Regional Medical Center, Palm Springs, USA; 2 Medicine, University of New England, Biddeford, USA; 3 Medicine, Ross University School of Medicine, Bridgetown, BRB; 4 Medicine, Western University of Health Sciences, Pomona, USA; 5 Medicine, Chapman University, Orange, USA; 6 Neurosurgery, University of Colorado, Boulder, USA

**Keywords:** lumbar arthroplasty, spinal-fusion, lumbar spinal stenosis (lss), preserve motion, degenerative spine disease, spinal decompression, dynamic motion, lumbar-fusion, back pain, chronic low back pain (clbp)

## Abstract

Degenerative disease of the lumbar spine commonly develops with age and can cause debilitating pain or neurologic deficits. When minimally invasive treatments and pain management interventions fail to provide relief, the traditional treatment has consisted of decompression surgery followed by the possible need for lumbar fusion. A mechanical implant device, known as a Total Posterior Spine (TOPS) System, has been introduced as a potential dynamic alternative to fusion surgery following decompression. The device is a dynamic posterior arthroplasty via pedicle screw insertion that maintains mobility, flexibility, and range of motion by providing multiaxial, three-column stabilization. While currently approved for use in Europe, the device is undergoing clinical trials in the United States to determine efficacy and potential complications. This paper provides a comprehensive narrative review of this technique's mechanism, early clinical outcomes, and considerations for patient selection. A review of the literature identified both positive results and adverse effects. While TOPS' use shows excellent potential, additional prospective trials are needed to determine this system's long-term complications.

## Introduction and background

Lumbar spinal stenosis (LSS) narrows the central canal and can be associated with spondylolisthesis, translating one vertebral segment on another [1]. While both have a wide array of possible etiologies, they are common sequelae of progressive lumbar degeneration, propagating significant functional restriction, instability, and locoregional or radicular pain [2]. Operative management for these disorders typically consists of neural decompression via laminectomy or facetectomy and with or without arthrodesis [3]. Surgical care is more effective than conservative treatment for pain and functional outcomes, and given the increasing incidence of back pain, its use will continue to expand [4,5].

The primary anatomic mechanism for dynamic spinal stability and functional mobility in the thoracic and lumbar spine rests on the posterior ligamentous complex (PLC) integrity and its associated bony structures. This comprises the supraspinous ligaments, interspinous ligaments, ligamentum flavum, and facet joints, as well as the laminae, spinous processes, and transverse processes [6]. It is thus favoured to preserve these structures in surgery; although this is not always possible. During decompressive laminectomy or facetectomy, some of these elements (most commonly the supraspinous and interspinous ligaments and the laminae) may be sacrificed to gain adequate exposure and relieve nerve root pressure [7].

Class II and III evidence show that fusion-decompression is superior to decompression alone for clinical and radiological outcomes in LSS and/or spondylolisthesis surgery, but this remains somewhat controversial [1, 8, 9]. While the techniques, approaches, and available instruments have improved, concerns remain regarding spinal stability, complications, and joint mobility [10]. Decompression alone can lead to spinal instability secondary to disruption of the PLC and posterior bony spine, while concurrent fixation can decrease mobility [11,12]. However, decompression-fusion patients have greater blood loss, higher reoperation rates, and longer operative times and admissions [1].

The Total Posterior Spine (TOPS) System (Premia Spine, Philadelphia, USA), first developed in 2003, provides an alternative. It aims to avoid spinal fusion drawbacks while achieving adequate decompression, preserving multiaxial stability, and restoring near-anatomic mobility. The TOPS System is an active single-level prosthesis wedged between two pedicle screw-fixated titanium plates. It allows for side bending, axial rotation, and flexion/extension range of motion while limiting sagittal translation [13]. It is currently indicated for LSS, spondylolisthesis, and facet arthrosis and has been increasingly proven safe and effective for these conditions [13-15]. Despite this encouraging evidence, it continues to be an experimental device and, thus, further high-quality studies are warranted. We aim to provide an updated and comprehensive review of the techniques, outcomes, and patient selection considerations in utilising the TOPS System.

## Review

Patient selection

Symptoms

Patients suffering from chronic low back pain, leg pain, or buttock pain may qualify as candidates to utilise the TOPS system for a total posterior spinal arthroplasty [16]. Specifically, patients may also complain of leg pain while walking or standing for a short period, likely indicating possible neurogenic claudication [13]. Symptoms are graded using the Visual Analogue Scale (VAS) and the Oswestry Disability Index (ODI) to determine each patient's candidacy; with scores of at least 40/100 at baseline for each to qualify for consideration. Patients must have also failed at least six months of conservative therapy for their symptoms, including but not limited to anti-inflammatory medications, physical therapy and epidural or facet joint injections [17].

Indications

Indications for patients to receive the TOPS procedure include diagnoses of lumbar spinal stenosis, spondylolisthesis, retrolisthesis and facet arthropathy [13, 17-19]. Before selection, each patient is investigated with imaging including computed tomography (CT) scan, magnetic resonance imaging (MRI), X-ray (including flexion/extension/neutral), and to a lesser extent CT myelogram (if unable to have MRI performed) to identify the pathology and anatomical location for which the procedure will take place [13]. Patients requiring decompression or fusion at a single vertebral level qualify for consideration; lumbar vertebra L2-L5 are good locations; however, most have taken place between L4-L5 [13]. Notably, previous surgeries including laminectomy, laminotomy, foraminotomy and discectomy do not exclude a patient from the candidate selection so long as no instrumentation has been implanted.

Contraindications

Patients who have undergone any prior spinal fusion surgeries with or without instrument implantation are contraindicated for the surgery since the intent is to maintain dynamic motion. Patients suffering from a grade II or higher spondylolisthesis or a spinal pathology involving more than one vertebra results are not ideal candidates for the procedure. Furthermore, patients with scoliosis of more than 10 degrees, back pain of unknown etiology and morbid obesity >40BMI result in a contraindication.

Technique

The TOPS device is a unitary implant consisting of two titanium plates with an interlocking flexible articulating core. It has metal arms that connect horizontally to pedicles with four polyaxial pedicle screws. This device can be implanted after a standard decompression via removal of the lamina and medial facets. The implant facilitates mobility in flexion, extension, and lateral bending, but provides stability in axial rotation [20].

To begin the TOPS procedure, the patient is appropriately positioned in the prone fashion on a Jackson frame in neutral lordosis. Four-poster or double roll padding configuration can be used depending on operator preference. Positioning should ensure that the patient's abdomen is free and recreates lumbar lordosis [13]. A standard midline incision is carried out to the lumbodorsal fascia measuring approximately 6-12 cm [14]. To expose posterior elements, bovie electrocautery is used for the subperiosteal dissection to elevate the musculoligamentous complex away from the posterior elements' spinous processes [13]. Exposure and retraction of the paravertebral musculature are extended only to the lateral aspect of the facet complex. It is unnecessary to expose the transverse processes in this procedure fully. The operator can preserve the capsule and muscular attachments proximate to the superior facet complex. After adequate exposure, the spinal canal's decompression is achieved with the removal of lamina and facet joints. Unroofing neural foramina do bilateral laminectomy and total functional facetectomy via the removal of both articular processes. The degree of bone, synovium, and ligamentum flavum resection may vary depending on the specific pathologic features in each case [13]. A Woodson elevator may be used to assess for appropriate decompression of the thecal sac and nerve roots. After decompression, a trial template is utilised to ensure bone excision is adequate for the prosthesis's accommodation.

Subsequently, pedicle screw placement is considered. To achieve maximal triangulation of the final screws, special attention is paid to attaining a more lateral-to-medial vector for the pedicles' cannulation. Intraoperative fluoroscopy can be employed to aid in the accurate positioning of screws. A pendulum-type guide wire helps to assure the trajectory of the pedicle screws in the axial plane within the range of the polyaxial tulip head screws for the four arms' geometrical constraints of the implant. To attain the maximal pullout strength of pedicle screws, triangulation of screw trajectories is done, and the largest anatomically feasible screw size is used. The cannulated screws provided with the TOPS system are selected to instrument the pedicles. After instrumentation, the dorsal height of the pedicle screws is adjusted with a two-part alignment gauge used to make sure that they are in the same coronal plane. Screw extension sleeves can be utilised to enable the implant's final attachment when the screw heads are difficult to access. The alignment gauge is additionally used as a trial to choose an appropriately sized TOPS implant. Sterile saline is then injected into a port on the implant to fill the central boot. Placement of the implant can then be done and is secured to the screw heads via set screws which are adequately tightened. Confirmation with fluoroscopy in biplanar fashion is done to validate the device position and screw trajectories, after which the extension sleeves can be removed if they were used. A suction drain can be inserted, and the surgical wound is closed in a standard layered fashion with particular attention given to the deep fascia closure. Ideally, patients can be mobilised on a postoperative day one and do not require external immobilisation [13,14].

Outcomes

TOPS has been shown in clinical studies to improve pain levels and maintain spinal range of motion (Table 1). Simultaneously, the TOPS system preserves the natural biomechanics of the spine when assessing intervertebral disc strain and bulge. The Visual Analog Scale (VAS), Oswestry Disability Index (ODI), and Short Form 36 (SF-36) were all utilised to quantitatively measures changes in pain and quality of life as a result of the implant. X-rays, CT's, and MRI's were additionally utilised to evaluate adverse events or unintended changes to the implant.

**Table 1:** Summarization of published outcomes.

Author and Year Published	Outcome
Wilke, H. et al. 2006 [20]	The TOPS implant restores range of motion (ROM) in side-bending and axial rotation, and 85% of flexion and extension ROM when compared to a pre-procedural intact lumbar segment. TOPS system maintains the physiological loading of the intervertebral disc in addition to the natural biomechanical posterior stabilization of the lumbar spine.
McAfee, P. et al. 2007 [13]	Utilized 100mm Visual Analog Scale (VAS), Oswestry Disability Index (ODI), and Radiographs to measure outcomes. Mean ODI decreased by 41% at 1 year post-operation, VAS decreased by 76mm at the 1 year post-operation. Radiographs showed preserved lumbar motion and disc height with no evidence of implant dysfunction, migration, or adverse events; Patient’s clinical status improved significantly post-procedure.
Heuer, F. et al. 2012 [21]	The TOPS system implant restored side-bending and axial rotation ROM, as well as 82% of the ROM in flexion/extension. Side-bending was shown to have a slight increase in ROM compared to pre-implantation. TOPS implant resulted in intervertebral disc (IVD) bulge and strain similar to physiological condition.
Anekstein, Y. et al. 2015 [14]	Follow up at 6wks, 3mos, 6mos, 1,2,3,7 years post-procedure VAS for back pain decreased from 56.2 preop to 12.5 at 6wks, 13.7 at 1yr, 3.6 at 2yrs, 19 at 7 yrs. VAS for leg pain decreased from 83.5 preop to 13 at 6wks, 9.2 at 1 yr, 3.6 at 2 yrs, 8.8 at 7 yrs. ODI decreased from 49.1 preop to 13.5 at 6wks, 8.6 at 1 yr, 3.3 at 2 yrs, 7.8 at 7 yrs. SF-36 (short form 36) increased from 43.2 preop to 69.9 at 6 wks, 80.2 at 1 yr, 82.8 at 2 yrs, 74.8 at 7 yrs. Radiographs did not show fusion or loosening of screws at 7 yrs follow-up. Additionally, they showed mobility in the implant with flexion/extension and a mean decrease in flexion/extension ROM from 6.1^o^ preop to 3.6 at 3mos, 5 at 1 yr, and 4.8 at 7 yrs No incidence of spondylolisthesis or stenosis adjacent to stabilization at 7 yrs. 4 patients experienced fluid buildup in the adjacent segment facet joint space. Disc degeneration at 2^nd^, 3^rd^, or 4^th^ level above index level was seen in 3 patients. 1 device failure out of 10 converted to a spinal fusion at 6 wks post TOPS system placement.
Smorgick, Y. et al. 2019 [15]	11 year follow-up of 2015 study [14]. Back pain VAS was 56.2 preop and decreased to 14 at 11 yrs Leg pain VAS was 83.5 preop and decreased to 17 at 11 yrs ODI was 49.1 at preop and decreased to 16 at 11 yrs MRI in one patient at 11 years postop demonstrated asymptomatic stenosis adjacent to stabilized segment. No evidence of progression to spondylolisthesis in any of the 10 cases at 11 year follow-up.

Table 1 also details multiple studies that showed no adverse fusion, loosening, or migration of the TOPS system. Only one patient required a spinal fusion after the TOPS system failed three months post-surgery [14]. Anekstein et al. and the Smorgick et al. conducted long-term prospective trials with detailed results regarding outcomes [14,15]. They showed that the TOPS system improved lower back and leg pain, with the greatest pain improvement at six weeks post-procedure. Furthermore, the pain relief continued at the 11-year post-procedure mark. However, the studies summarised in Table 1, show that the TOPS implant described several adverse events when patients participated in long term follow up. Some of these negative outcomes included fluid buildup in the adjacent segment facet joint space, disc degeneration at second, third, or fourth level above index level, and adjacent level spinal stenosis. The theorised source of the adverse outcomes is that the surgically produced dynamic motion at the spinal level of interest contributes additional strain on the spinal levels above and below. Spinal surgeons must weigh the risks and benefits of this procedure before introducing it to patients. Patients must be educated on such risks during the informed consent process. Overall, there is a lack of long-term follow-up studies. Continued investigations and randomised controlled prospective trials are needed to determine outcomes and discuss those outcomes with patients intelligibly.

## Conclusions

The findings of this review suggest that while there is strong potential behind the TOPS implant ability to reduce pain while maintaining spinal ROM, further research is warranted to establish the long-term potential adverse effects. Additionally, further research should aim to develop, in greater detail, the possible causes of adverse effects based on patient history to identify additional contraindications.
